# jmzTab: A Java interface to the mzTab data standard

**DOI:** 10.1002/pmic.201300560

**Published:** 2014-04-29

**Authors:** Qing-Wei Xu, Johannes Griss, Rui Wang, Andrew R Jones, Henning Hermjakob, Juan Antonio Vizcaíno

**Affiliations:** 1 European Molecular Biology Laboratory, European Bioinformatics Institute (EMBL-EBI), Wellcome Trust Genome CampusHinxton, Cambridge, UK; 2 Department of Computer Science and Technology, Hubei University of EducationWuhan, China; 3 Division of Immunology, Allergy and Infectious Diseases, Department of Dermatology, Medical University of ViennaAustria; 4 Institute of Integrative Biology, University of LiverpoolLiverpool, UK

**Keywords:** Bioinformatics, Data standard, Java application programming interface, Proteomics Standards Initiative

## Abstract

mzTab is the most recent standard format developed by the Proteomics Standards Initiative. mzTab is a flexible tab-delimited file that can capture identification and quantification results coming from MS-based proteomics and metabolomics approaches. We here present an open-source Java application programming interface for mzTab called jmzTab. The software allows the efficient processing of mzTab files, providing read and write capabilities, and is designed to be embedded in other software packages. The second key feature of the jmzTab model is that it provides a flexible framework to maintain the logical integrity between the *metadata* and the table-based sections in the mzTab files. In this article, as two example implementations, we also describe two stand-alone tools that can be used to validate mzTab files and to convert PRIDE XML files to mzTab. The library is freely available at http://mztab.googlecode.com.

In the last decade, several vendor-neutral standard data formats were developed by the HUPO Proteomics Standards Initiative (PSI, http://www.psidev.info), to promote data sharing and software development in the field. So far, most of the released data standards related to MS-based proteomics are XML-based: mzML (for MS data) 1, mzIdentML (for peptide and protein identifications) 2, mzQuantML (for quantification data) 3, and TraML (for transition lists in targeted proteomics approaches) 4. During the development of mzIdentML and mzQuantML the main focus was put on providing an accurate and comprehensive representation of the data. This resulted in relatively complex XML schemas, which in some cases, can make it difficult for data consumers to access the information. In addition, no standard format existed for MS-based metabolomics results. To overcome these issues, the mzTab standard (J. Griss et al., paper submitted) was recently developed as a flexible tab-delimited file format, to report proteomics and metabolomics results, including both identification and quantification data. At the time of writing, the first stable version (v1.0) of the standard is about to be released (see the updated specification document at http://mztab.googlecode.com).

mzTab has a flexible design which allows the reporting of identification and quantification results at different levels, ranging from a simple summary or subset of the complete information (e.g., the *final results*) up to fairly comprehensive representation of the results including the experimental design. Many data consumers are only concerned about having access to the *final results* of a study in an easily accessible format that is compatible with tools like Microsoft Excel® or the R programming language (R Core Team, http://www.R-project.org), among others. For this reason, mzTab is also aimed to make MS proteomics and metabolomics results available to the wider biological community, beyond the field of MS.

An mzTab file can have up to five different sections: *metadata*, *protein*, *peptide*, *psm* (peptide spectrum match), and *small molecule*. In addition, it can reference the corresponding mass spectra in the relevant external files. There are two types of mzTab files: ‘‘Identification’’ (including peptide, protein, and/or small molecule identifications) and ‘‘Quantification’’ (used for quantification results, and optionally may contain identification results as well). In addition, there are two levels of detail (called ‘‘mode’’) of reporting data: ‘‘Summary’’ and ‘‘Complete.’’ The ‘‘Summary’’ mode can be used to report the *final results* of a study, for example, reporting data averaged from different replicates. The ‘‘Complete’’ mode is used if detailed experimental information coming from each individual assay/replicate is provided. The experimental design is modeled in a similar way to mzQuantML, including the elements ‘‘study_variable’’, ‘‘assay’’, ‘‘ms_run’’, and ‘‘sample’’ (see mzTab specification document for more details).

There are already several tools implementing mzTab. For example, mzTab is in use in the OpenMS Proteomics Pipeline 7 and fully supported by the MSnbase R/Bioconductor package 8. Also, as the first nonproteomics tool, the LipidDataAnalyzer 9 supports the export of mzTab files, including quantitative information extracted from lipidomics MS data. In addition, two prominent data resources, the PRIDE database (for MS-based proteomics data) 10 and MetaboLights (for metabolomics data) 11 make use of the new standard and are planning to use it heavily in the near future.

Here we introduce jmzTab, an open source Java application programming interface (API) for reading, writing, and validating mzTab files. This API greatly simplifies accessing the information included in mzTab files, thereby promoting its use and facilitating its support in third party software. Analogous Java libraries were developed before for other PSI standards, such as jmzML 12, jmzIdentML 13, jmzQuantML 14 or jTraML 15. The API is released under the permissive Apache License 2.0, and the source code is available at http://mztab.googlecode.com. In addition, as example implementations of the library, we describe two stand-alone tools supporting validation and conversion functionality for the format. Additionally, mzTab example files that can be used to test the API are available at https://code.google.com/p/mztab/wiki/ExampleFiles.

jmzTab is structured in a three-layer architecture: (i) the *Core Model Layer*, a lightweight independent implementation for maintaining the integrity between the different sections in the file; (ii) the *Enhancement Utilities Layer*, which provides parsing, validation, and conversion functionality; and (iii) the *Standalone Application Layer*, which constitutes the centralized graphical user interface (GUI) and command line entry point for the conversion and validation functionality. The main classes of the *Core Model* are displayed as an UML (Unified Modified Language) diagram in Fig.1. Detailed documentation about how to use the API can be found at https://code.google.com/p/mztab/wiki/jmzTab2 and at http://mztab.googlecode.com/svn/jmztab/trunk/docs/index.html.

**Figure 1:**
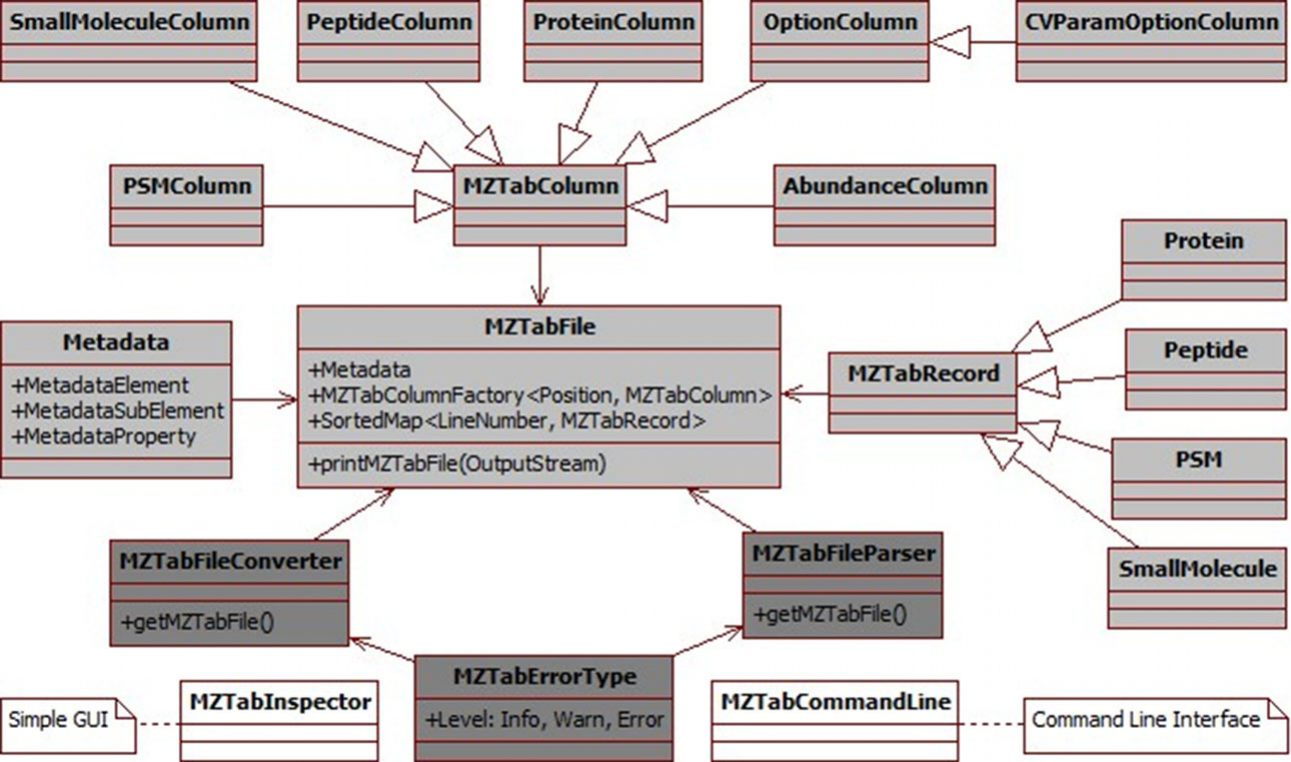
Simplified UML diagram of the jmzTab API. The classes are structured in three different layers: the *Core Model Layer* (highlighted in gray), the *Enhancement Utilities Layer* (dark-gray), and the *Standalone Application Layer* (white). See main text for more details. The diagram does not include all the classes and methods.

The main principle behind jmzTab *Core Model*’*s* design is to provide an independent, light-weight architecture to simplify the integration of the library into different proteomics/metabolomics software applications. In fact, the model can be integrated into external applications without the need for any other third-party packages. The second key feature of the *Core Model* is the use of a flexible framework to keep the logical data integrity between the *metadata* and the table-based sections (including the *protein*, *peptide*, *psm*, and *small molecule* sections). In the jmzTab *Core Model*, the *MZTabFile* class is the central entry point to manage the internal relationships among the different sections in the file. It contains three key components: (i) *Metadata*, which is a mandatory meta model that provides the definitions from the dataset included in the file; (ii) *MZTabColumnFactory*, a factory class that can be used to generate stable *MZTabColumn* elements, and to add different optional columns dynamically (e.g., protein and peptide abundance related columns). The *Metadata* and *MZTabColumnFactory* constitute the framework for the *MZTabFile* class; and (iii) Consistency constraints among the different sections of the model. For example, the *MZTabFile* class supports the iterative modification of the elements ‘‘study_variable’’, ‘‘ms_run’’, ‘‘assay’’, and ‘‘sample’’ assigned numbers (1−n) and its concrete location in the file, maintaining the internal consistency between the *metadata* section and the optional elements in the table-based sections. These methods are particularly useful when information from different experiments (e.g., from different MS runs) is condensed into a single mzTab file.

As mentioned above, in addition to the *Core Model*, the classes included in the *Enhancement Utilities Layer* provide mzTab parsing, validation, and conversion related functionality:

- Parsing and validation: mzTab files can be validated to ensure that they comply with the latest version of the format specification. The process includes two steps. First of all, the basic model architecture is created, including the metadata section and the generation of the table column headers. The second step is the validation of the column rows. The class *MZTabFileParser* is used to parse and validate the mzTab files. If the validation has completed, an *MZTabFile* model will be generated. A series of messages are then reported, which can help to diagnose different types of format-related and/or logical (reporting errors related to the logical relationships among the different sections in a file) errors. At the time of writing, there are about sixty types of error messages (https://code.google.com/p/mztab/wiki/jmzTab2_message). Each validation message has a unique identifier and is classified in three levels: *Info*, *Warn*, and *Error*, according to the requirements from the specification document.
- Conversion from PRIDE XML (internal XML format used by PRIDE) files to mzTab: The library supports the one-to-one conversion from PRIDE XML to mzTab. This functionality is provided since the PRIDE team is starting to make all proteomics results available in this format. In addition, by extending the *ConvertProvider* class, the current model can be used for the conversion of third-party format files. Converters from mzIdentML and mzQuantML into mzTab are being developed at present making use of this capability, for example, as part of the mzidLibrary 16. The objective is that the researchers will always be able to access the information in PRIDE through the mzTab format, independently of the format used for the data submission.

As example implementations, two stand-alone tools were developed. Both tools are included in a zipped file, available to download at https://code.google.com/p/mztab/downloads/list.

- *mzTabGUI*, a desktop application which provides mzTab validation functionality and conversion from PRIDE XML files to mzTab, in two different tabs (Fig.2). After the conversion, the tool additionally performs an automatic validation of the mzTab file.
- *mzTabCLI*, a command line interface (CLI) which provides a more flexible way of processing mzTab files in a batch mode. It also includes validation and conversion functionality.

**Figure 2:**
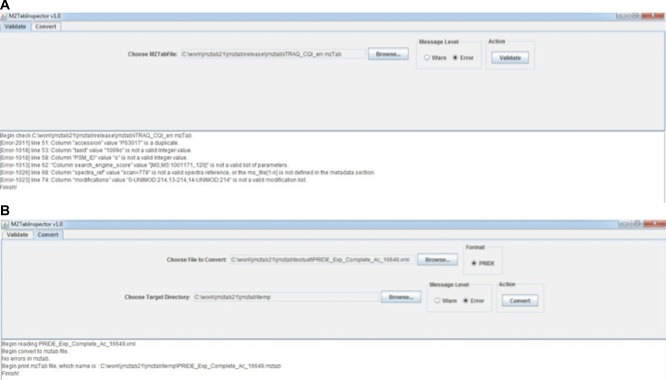
Screenshots of the *mzTabGUI* tool. (A) Example of validation report. Six error messages are output in the console panel. (B) Example of conversion of one PRIDE XML file to mzTab.

In addition, it is important to highlight that jmzTab is already integrated and used in other applications such as the LipidDataAnalyzer (http://genome.tugraz.at/lda/), and in an mzQuantML to mzTab converter included in the *mzq-lib* library (https://mzq-lib.googlecode.com/). The two stand-alone tools described above also play an important role in parsing, converting, and validating mzTab files in these projects. Detailed documentation about how to use the API can be found at https://code.google.com/p/mztab/wiki/jmzTab2 and at http://mztab.googlecode.com/svn/jmztab/trunk/docs/index.html.

## Conclusions

We have presented the open-source library jmzTab to support the PSI's new mzTab standard format. The API follows the design principles used in other existing analogous APIs for other PSI standards such as jmzML or jmzIdentML. Since jmzIdentML and jmzML are focussed around XML formats, jmzTab had to be developed from scratch. In addition, we present two stand-alone tools, which make use of the API. It is planned that the mzTab format will be heavily used in prominent resources such as PRIDE and MetaboLights. Therefore, it is expected that jmzTab will be one of the essential pieces of software to facilitate data provision and data access to both resources.

## Abbreviations

API: Application Programming Interface
PRIDE: PRoteomics IDEntifications (database)
PSI: Proteomics Standards Initiative
XML: eXtensible Markup Language
